# Ginsenoside Rg1 ameliorates renal ischemia-reperfusion injury by inhibiting FABP1-regulated Nrf2/HO-1 pathway

**DOI:** 10.1080/0886022X.2026.2650261

**Published:** 2026-05-03

**Authors:** Xiaodong Chang, Lu Zhang, Guangyi Zheng, Hongyu Zhang, Hen Xue, Wanyan Feng

**Affiliations:** Department of Nephrology, Ya’an People’s Hospital, Yaan, P.R. China

**Keywords:** Renal ischemia-reperfusion injury, Ginsenoside Rg1, Nrf2/HO-1 pathway, fatty acid-binding proteins 1

## Abstract

Ginsenoside Rg1 (G-Rg1) can effectively ameliorate lipopolysaccharide-induced renal injury. The impact and mechanism of G-Rg1 in renal ischemia-reperfusion (I/R) injury are not yet understood. This study aimed to examine the role and mechanism of G-Rg1 in kidney I/R injury. The renal I/R injury mice and mouse kidney cells were applied as renal I/R injury models. Researchers analyzed the functions and mechanisms of G-Rg1 using techniques like cell proliferation, apoptosis, clone formation assay, ELISA, HE staining, immunohistochemical staining, Immunofluorescence staining, qRT-PCR, and Western blot analysis. Our findings indicate that G-Rg1 pretreatment protects against renal I/R injury by lowering serum creatinine and urea nitrogen levels, mitigating histological damage and apoptosis, and reducing inflammation and oxidative stress. These beneficial effects were accompanied by the suppression of fatty acid binding protein 1 (FABP1) and heme oxygenase-1 (HO-1) expression and the promotion of nuclear factor erythroid 2-related factor 2 (Nrf2) nuclear translocation. However, the therapeutic effect of G-Rg1 was inhibited by FABP1 overexpression. The mechanism by which G-Rg1 ameliorates renal I/R injury may be related to the inhibition of FABP1 expression and thus regulation of the Nrf2/HO-1 pathway.

## Introduction

Acute kidney injury (AKI) is a worldwide health issue because of its high rates of illness, death, and treatment expenses [1]. Ischemia-reperfusion (I/R) injury is a pathological process characterized by insufficient or interrupted blood supply to tissues, where subsequent restoration of blood flow (reperfusion) paradoxically exacerbates tissue injury and dysfunction rather than restoring normal function [2]. Renal I/R injury is the main cause of AKI [3], which can be induced by renal transplantation, renal oncologic surgery, renal vascular surgery, etc [4]. Inflammatory response, oxidative stress, nitric oxide, and activation of the complement system are currently considered to play important roles in the pathophysiology of renal I/R [5,6]. Previous experimental studies have identified several drugs that may be protective against renal I/R in response to these mechanisms [7,8]. However, to date, no effective strategies have been established for the alleviation of renal I/R in clinical practice.

Ginsenoside Rg1 (G-Rg1) is one of the major active ingredients in Ginseng [9]. G-Rg1 has demonstrated efficacy in treating multi-organ diseases, including hepatic, cardiac, nervous system, and renal disorders [9,10]. Its anti-aging, anti-tumor, and anti-diabetic effects have also been continuously discovered [11,12]. In terms of organ protection, G-Rg1 treatment effectively attenuated lipopolysaccharide- and d-galactose- induced acute liver failure, and reduced necrosis and apoptosis of hepatocytes [13]. It has also been shown that G-Rg1 treatment reduced reactive oxygen species (ROS) production in hypoxic/reoxygenated rat cardiomyocytes and restored cardiac function in the infarcted area [14]. In addition, G-Rg1 was effective in ameliorating sepsis or lipopolysaccharide-induced renal injury [15,16]. However, the role of G-Rg1 in renal I/R injury and its mechanism have not been clear.

The main biological function of fatty acid-binding protein 1 (FABP1) is to regulate fatty acid metabolism, and FABP1 expression was also detected in renal tubular cells and alveolar epithelial cells [17]. Silencing of FABP1 reduced the expression of TNF-α, IL-6, and heme oxygenase-1 (HO-1) thereby ameliorating hepatic inflammation and oxidative stress [18]. Studies have shown that FABP1 also has a protective effect against oxidative stress in the kidney and reduces tubulointerstitial and glomerular injury and nephrotoxicity [19]. Additionally, the nuclear factor erythroid 2-related factor 2/heme oxygenase-1 (Nrf2/HO-1) pathway might contribute to renal injury by influencing inflammation, oxidative stress, and apoptosis [20,21]. However, whether G-Rg1 affects the Nrf2/HO-1 signaling pathway through the regulation of FABP1 and thus improves renal I/R is unknown.

Therefore, this study will analyze from *in vivo* and *in vitro* experiments whether G-Rg1 affects the Nrf2/HO-1 signaling pathway by modulating FABP1 expression and thus improves renal I/R, which provides insights into the prevention and treatment of renal I/R by G-Rg1.

## Materials and methods

### Animal experiments

Twenty-five male C57BL/6 mice, 8 weeks, weighing 20–25 g, were purchased from Sichuan Charles River (License No. SCXK (Chuan) 2023-0040). Mice were conditioned to live in an environment with a 12-h light/dark cycle, a relative humidity between 30% and 70%, and temperatures ranging from 20 °C to 26 °C, with unrestricted access to food and water. Following a one-week acclimatization period, the mice were randomly assigned to the sham group (sham, *n* = 5), renal ischemia-reperfusion injury model group (Model, *n* = 5), G-Rg1 low dose group (G-Rg1-L, *n* = 5), G-Rg1 medium dose group (G-Rg1-M, *n* = 5), G-Rg1 high dose group (G-Rg1-H, *n* = 5), The sample size is calculated based on statistical power. The trial was approved by the Experimental Animal Ethics of West China Hospital of Sichuan University (No.20240603001). Following completion of the experiments, animals were anesthetized *via* intraperitoneal injection of sufentanil combined with dexmedetomidine (10 mL/kg, Hengrui Medicine, Jiangsu, China). The animals were executed after inhalation of carbon dioxide to obtain kidney tissue and blood.

Before the mice were modeled, the G-Rg1-L, G-Rg1-M, and G-Rg1-H groups were gavaged with 1 mg/kg, 5 mg/kg, and 10 mg/kg of G-Rg1 (CAS No: 22427-39-0, DESITE, Chengdu, China), once a day for one week, respectively. The sham group and the model group were gavaged with equal amounts of saline. Subsequently, the mice were anesthetized with intraperitoneal injection of Zoletil (40 mg/kg, Virbac, Carros, France) combined with dexmedetomidine (0.1 mg/kg, Xinhua Pharmaceutical Co., Ltd., Shandong, China). Then, a small incision was made through the midline of the abdomen and the right kidney was exposed, nephrectomy was performed, and then the left kidney was exposed and the renal artery was ligated with a silk thread, and the kidneys were reperfused after 45 min of ischemia, and the kidneys could be observed to change from dark purple to red. Mice in the sham group underwent all surgical procedures except ligation. HE staining of renal tissue revealed patchy and focal necrosis of tubular epithelial cells; destruction of the tubular basement membrane was observed in severe lesions, indicating successful modeling. Serum and kidney tissue samples were collected 180 min after reperfusion. The renal coefficient was calculated as (kidney wet weight/body weight)×100%.

### Cell experiments

The mouse kidney cells tubular cell mouse kidney-1 (TCMK-1) (icell, Shanghai, China) was cultured in Dulbecco’s modified eagle medium/nutrient mixture F-12 (DMEM/F12) medium (Procell, Wuhan, China) containing 10% fetal bovine serum at 37°C with 5% CO_2_. Cells were passaged when the cell fusion reached 85% or more. Setting the cell grouping (i) Control + G-Rg1 (0, 25, 50, 75, 100, 125, 150 μM), I/R (*in vitro* ischemia/reperfusion model) + G-Rg1 (0, 25, 50, 75, 100, 125, 150 μM); (ii) Control group, I/R group, I/R + si-NC group (negative control), I/R + si-FABP1 group (FABP1 low expression); (iii) Control group, I/R group, I/R + Ov-NC group (negative control), I/R + Ov-FABP1 (FABP1 over-expression); (iv) I/R group, I/R + G-Rg1 group (100 μM), I/R + G-Rg1 + Ov-NC (negative control) group, I/R + G-Rg1 + Ov-FABP1 (FABP1 over-expression) group.

The G-Rg1 intervention group was cultured with medium containing the corresponding concentration of G-Rg1 for 12 h before I/R modeling. Then the medium was changed to hypoxia solution and then placed in 94% N_2_, 5% CO_2_, and 1% O_2_ hypoxic environment for 4h. After hypoxia, the medium was changed to DMEM/F12 containing 10% fetal bovine serum, and then placed in 74% N_2_, 5% CO_2_, and 21% O_2_ aerobic environment for 12h to make the *in vitro* ischemia/reperfusion (I/R) modeling. The Control group was under the condition of 74% N_2_, 5% CO_2_, and 21% O_2_ culture. In addition, the preparation of anoxic solution: 0.9 mmol/L NaH_2_PO_4_, 6.0 mmol/L NaHCO_3_, 1.8 mmol/LCaCl_2_, 1.2 mmol/L MgSO_4_, 98.5 mmol/L NaCl, 10.0 mmol/L KCl, 40 mmol/L sodium lactate, 20 mmol/L HEPES, pH = 6.8, pre-saturated with high purity N_2_ for 1h, the reoxygenated solution was the complete medium.si-NC, si-FABP1, Ov-NC, or Ov-FABP1 were produced by GenePharma (Shanghai, China). Cells were transfected with Lipofectamine^®^2000 transfection reagent (Invitrogen, Shanghai, China) before I/R modeling according to the above grouping. I/R modeling were performed as described above.

### Enzyme-linked immunosorbent assay (ELISA)

Serum levels of creatinine (Cr), urea nitrogen (BUN), superoxide dismutase (SOD), malondialdehyde (MDA), glutathione peroxidase (GSH-PX), and cellular activities of SOD. MDA, and GSH-PX were detected by ELISA, and analyzed strictly according to the ELISA kit (ZCI Bio, Shanghai, China).

### Hematoxylin-eosin (HE) staining

Kidney tissue sections (5 μm) were stained with hematoxylin for 20 min, hydrochloric acid alcohol differentiation for 10 s, put into warm water at 50 °C to return to the blue, stained with eosin for 5 min, transparent with xylene, and sealed with neutral gum, and digital trinocular camera microcamera system (BA400Digital, Motic, Xiamen, China) for image acquisition.

### Immunohistochemical staining

Kidney tissue slices, 5 μm thick, were deparaffinized, antigen retrieval was performed, endogenous peroxidase was inhibited with 3% hydrogen peroxide, then serum blocking was applied, and incubated overnight at 4 °C with the addition of primary antibody FABP1 (1:100, Servicebio, Wuhan, China). Apply the secondary antibody (HRP-labeled goat anti-rabbit, 1:100, Servicebio, Wuhan, China) and let it incubate at 37 °C for half an hour. DAB color development, re-staining with hematoxylin, sealing, and image capture using a digital trinocular camera microcamera system (BA400Digital, Motic, Xiamen, China), and Indica Labs’ Halo data analysis system, located in the USA, was used to assess the percentage of positive area (% DAB Positive Tissue) in each image.

### Immunofluorescence staining

Paraffin sections were deparaffinized by dewaxing solution (Jiangyuan industrial technology and trade corporation, Wuxi, China), immersed in repair solution and repaired in a microwave oven for 20 min, added with prepared dihydroethidium (DHE) probe solution (Beyotime, Beijing, China), and incubated at 37 °C for 30 min, protected from light. Subsequently, an anti-fluorescence quenching sealer was used to seal it. The sections’ images were taken using scanning and browsing software (OlyVIA, OLYMPUS, Japan), and the fluorescence intensity of each captured image was quantified using Image-J from the National Institutes of Health, USA.

### CCK-8 assay

After incubating the cells for 24 h, the medium was refreshed, and 10 μL of CCK-8 reagent (Biosharp, Guangzhou, China) was added to each well. The plates were kept in the dark for incubation, and cell viability was determined by measuring the absorbance at 450 nm using a microplate reader (ELx800, BioTek, USA).

### Flow cytometry assay

Cells and tissue samples were gathered, spun at 1000 r/min for 5 min, then resuspended in 100 μL of PBS, followed by the addition of 500 μL of 1 × Annexin V Binding Buffer (KeyGEN, Nanjing, China), and add 5 μL of Annexin VFITC (KeyGEN, Nanjing, China), mix gently, and incubate for 15 min at room temperature under the condition of avoiding light, add 5 μL of propidium iodide (PI, KeyGEN, Nanjing, China), and store the samples on ice, away from light, and then use a Cytoflex flow cytometer (Beckman Coulter, USA) to identify apoptosis within 60 min.

### Clone formation assay

The culture was terminated when a single cell grew to more than 50 cell clones and clones visible to the naked eye appeared in the 6-well plate as observed under the microscope. The supernatant was discarded, pure ethanol was added, fixed for 30 min, the fixative was removed, 0.1% crystal violet staining was performed for 20 min (Bomei, Hefei, China), air drying was performed, and photographs were taken to collect images. The number of clones with more than 50 cells was counted, and finally the number of clone formation was calculated.

### Quantitative real-time PCR (qRT-PCR)

Total RNA was extracted from the kidney tissue and cells using an ultra-pure RNA extraction kit (YEASEN, Shanghai, China), and 5 μL of RNA was taken to detect the integrity of RNA. The residual genomic DNA in the RNA was digested with a DNase I kit (Takara, Japan) and reverse transcription was performed using a reverse transcription kit (Takara, Japan). Amplification was performed using TB Green ^TM^ Premix Ex Taq^TM^ II (Takara, Japan). The sequences of the primers used are listed in Table 1. The relative expression of each target gene was quantified by the 2^-ΔΔCt^ method; β-actin was used as an internal control.

**Table 1.** Primer sequence.

Gene	Forward sequences (5′-3′)	Reverse sequences (5′-3′)
NGAL	CGCTACTGGATCAGAACATTTG	CTTGCACATTGTAGCTCTGTAC
KIM-1	CCTGCTGCTACTGCTCCTTGTG	CCACGCTTAGAGATGCTGACTTCC
FABP1	TTTCAAAGGCATAAAGTCCGTG	CTTGCTGACTCTCTTGTAGACA
IL-6	CTCCCAACAGACCTGTCTATAC	CCATTGCACAACTCTTTTCTCA
IL-10	TTCTTTCAAACAAAGGACCAGC	GCAACCCAAGTAACCCTTAAAG
TNF-α	ATGTCTCAGCCTCTTCTCATTC	GCTTGTCACTCGAATTTTGAGA
β-actin	CTACCTCATGAAGATCCTGACC	CACAGCTTCTCTTTGATGTCAC

NGAL: Neutrophil Gelatinase-Associated Lipocalin; KIM-1: Kidney Injury Molecule-1; FABP1: Fatty acid binding protein 1; IL-6: Interleukin-6; IL-10: Interleukin-10; TNF-α: Tumor Necrosis Factor-alpha; β-actin: beta-actin

### Western blot analysis

Cells and kidney tissue were lysed using radio-immunoprecipitation assay buffer (RIPA) cell lysis buffer, followed by centrifugation to obtain the protein supernatant. After adding SDS loading buffer, the samples were boiled. Proteins were separated by sodium dodecyl sulfate–polyacrylamide gel electrophoresis (SDS-PAGE) and transferred onto polyvinylidene fluoride (PVDF) membranes, which were then blocked with 5% skim milk powder at room temperature. After incubation with primary antibodies (anti-FABP1, 1:1000; anti-Bcl-2, 1:1000; anti-Bax, 1:1000; anti-cleaved-Caspase3, 1:1000; anti-HO-1, 1:5000; anti-Nrf2, 1:2000; anti-β-actin, 1:50000; anti-LaminB, 1:5000; the above antibodies were purchased from ABclonal (Wuhan, China)), washed with PBS three times, and goat anti-rabbit IgG (H + L) HRP (1:5000, Affbiotech, Nanjing, China) antibody was added for incubation. A luminous solution was added, and enhanced chemiluminescence (ECL) was used to observe the final results.

### Statistical analysis

GraphPad Prism 8 (GraphPad Software, San Diego, USA) was used to analyze the data, which are displayed as the mean ± SD. One-way ANOVA was used to compare the groups, and differences were deemed statistically significant if *P* values were below 0.05.

## Results

### Effect of G-Rg1 on the pathology and apoptosis of renal ischemia-reperfusion injury

**Figure 1.**
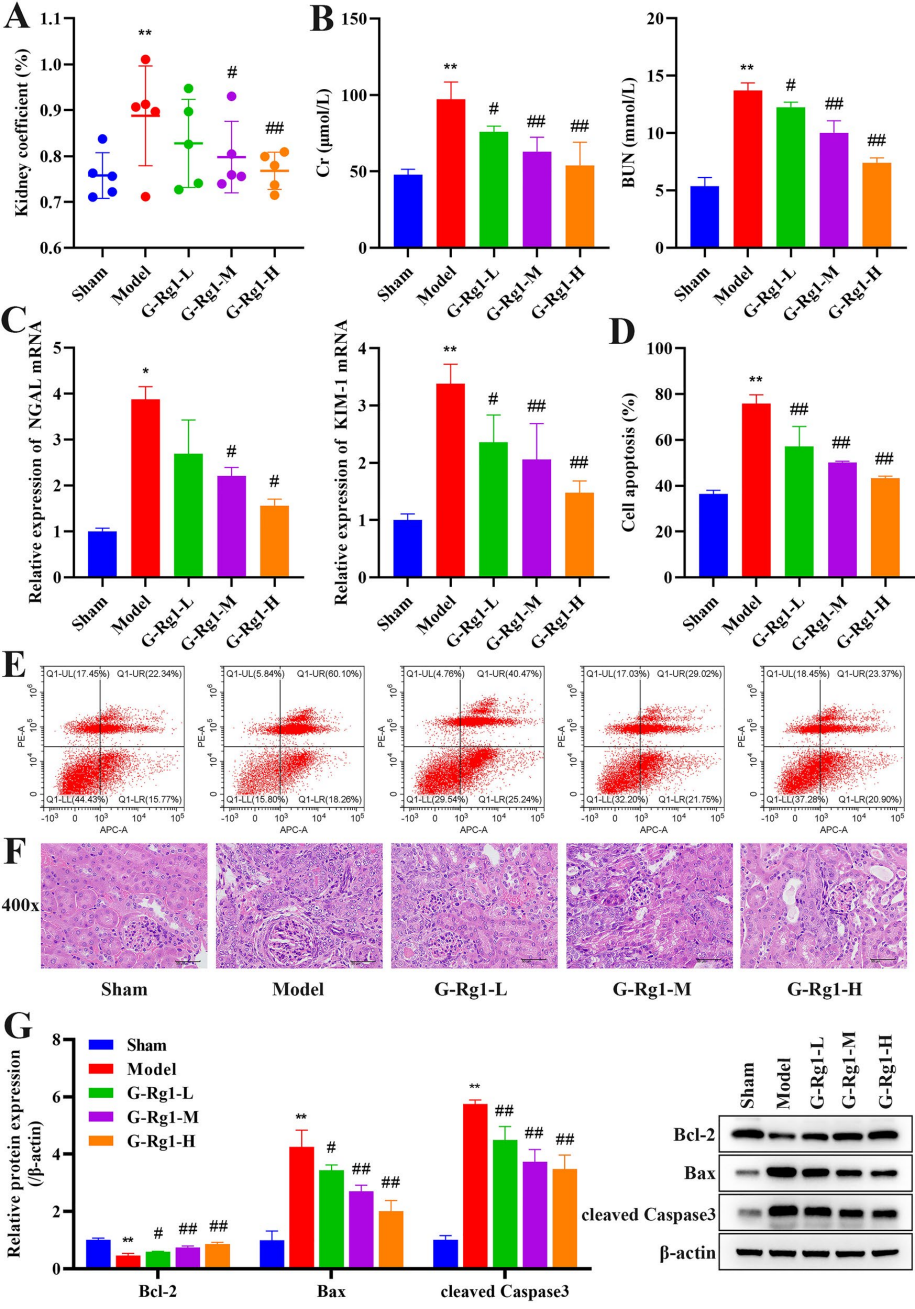
Effect of G-Rg1 on the pathology and apoptosis of renal ischemia-reperfusion injury. (A) Kidney coefficients for each group of mice. (B) Levels of Cr and BUN in the serum of mice. (C) The mRNA expression of NGAL and KIM-1 in mouse kidney tissues. (D) Apoptosis in mouse kidney tissue. (E) Apoptosis flow cytogram. (F) HE staining of kidney tissue (40×, Scale bar, 50 μm). (G) Western blot results of Bcl-2, Bax, and cleaved Caspase3 expression in kidney tissue, protein band calculated as a ratio relative to β-actin protein levels. The data are expressed as the mean ± SD. Compared with the sham group, *p < 0.05, **p < 0.01. Compared with the model group, #*p* < 0.05, ##*p* < 0.01.

First, as shown in Figure 1A and B, renal coefficient, Cr, and BUN were significantly increased in the model group compared with the sham group (*p* < 0.01), and the expression of renal injury markers NGAL and KIM-1 was significantly elevated in the model group compared with the sham group (Figure 1C, *p* < 0.05), and apoptosis was significantly promoted (Figure 1D and E, p<0.01), but G-Rg1-M and G-Rg1 -H was able to significantly inhibit the increase of the above indicators (*p* < 0.05). Pathological results showed that renal tubular epithelial cells of renal tissues in the model group were swollen, with nuclei protruding into the tubular lumen and with fibrous tissue hyperplasia, and G-Rg1 ameliorated the above pathological damage (Figure 1F). Moreover, compared with the sham group, the expression of Bcl-2 was significantly reduced, whereas the expression of Bax and cleaved Caspase3 was significantly increased in the model group (*p* < 0.01), and G-Rg1 treatment reversed the expression of Bcl-2, Bax, and cleaved Caspase3 (Figure 1G, *p* < 0.05), indicating that G-Rg1 ameliorated renal ischemia-reperfusion injury.

### Effect of G-Rg1 on oxidative stress and inflammation in mice with renal ischemia-reperfusion injury

**Figure 2.**
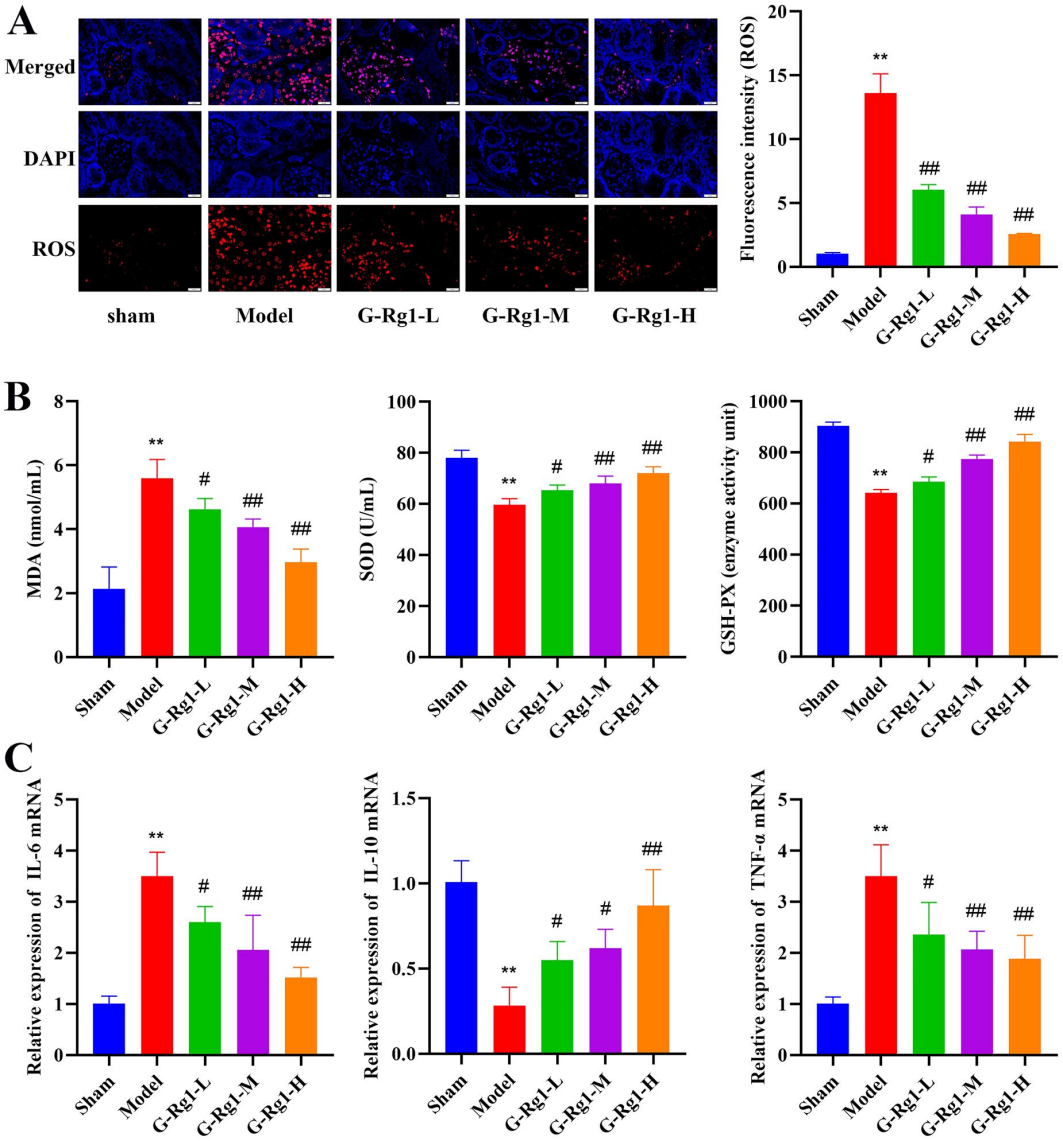
Effect of G-Rg1 on oxidative stress and inflammation in mice with renal ischemia-reperfusion injury. (A) Immunofluorescence staining for ROS in renal tissues (40×, Scale bar, 20 μm). (B) Activities of SOD, MDA, and GSH-Px in serum. (C) Expression of inflammatory factors IL-6, IL-10, and TNF-α in renal tissue. The data are expressed as the mean ± SD. Compared with the sham group, *p < 0.05, **p < 0.01. Compared with the model group, #*p* < 0.05, ##*p* < 0.01.

Compared with the sham group, the level of ROS in the renal tissues and the content of MDA in serum were significantly increased, whereas the activities of serum SOD and GSH-Px were significantly decreased in mice in the model group (Figure 2A and B, p<0.01). In addition, the expression of the inflammatory factors IL-6 and TNF-α in the renal tissues of mice in the model group was significantly increased, and that of IL-10 was decreased (Figure 2C, *p* < 0.01). G-Rg1 treatment improved the levels of the above factors in the model group (*p* < 0.05), showing that G-Rg1 inhibited the activation of oxidative stress and the levels of pro-inflammatory factors in renal ischemia-reperfusion injury.

### Effect of G-Rg1 on the expression of FABP1, HO-1, Nrf2 in mice with renal ischemia-reperfusion injury

**Figure 3.**
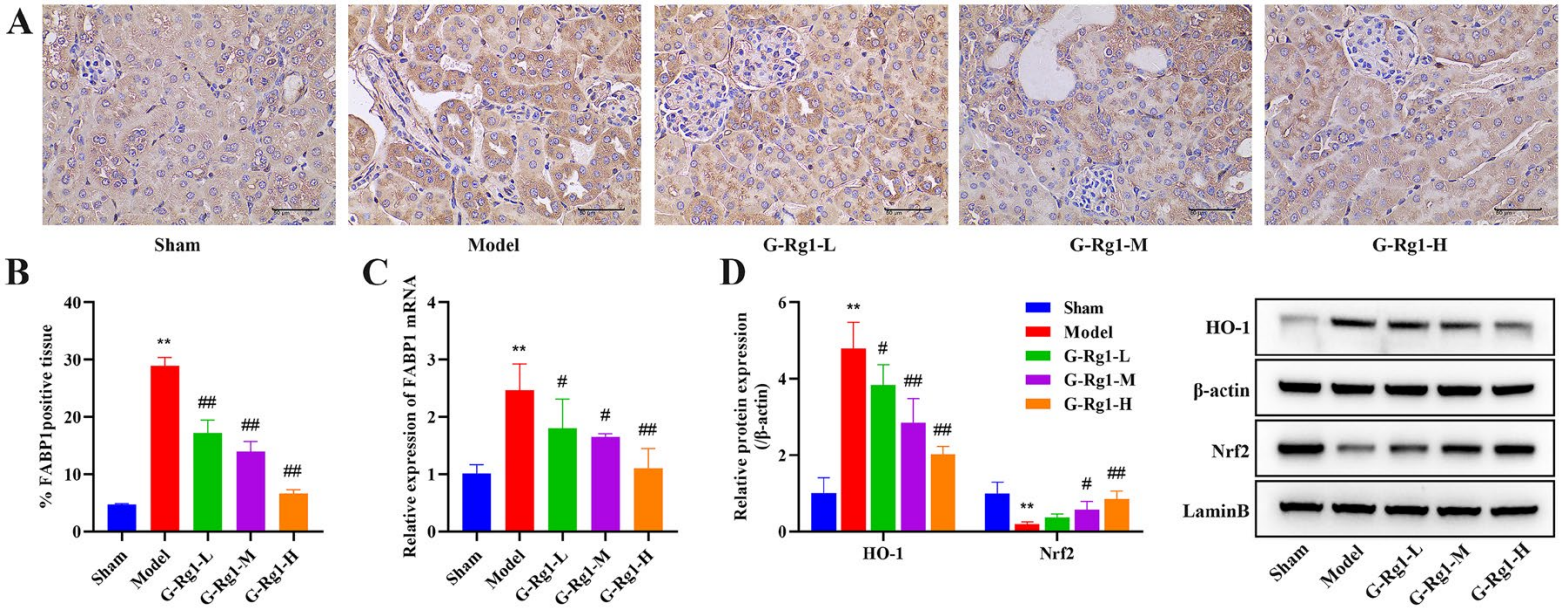
Effect of G-Rg1 on the expression of FABP1, HO-1, Nrf2 in mice with renal ischemia-reperfusion injury. (A) Immunohistochemical staining of FABP1 in renal tissue (40×, Scale bar, 50 μm). (B) Quantitative immunohistochemical staining of FABP1 in kidney tissue. (C) The mRNA expression of FABP1 in kidney tissue. (D) Western blot results of HO-1 and Nrf2 (nucleus) expression in kidney tissue, protein band calculated as a ratio relative to β-actin or LaminB protein levels. The data are expressed as the mean ± SD. Compared with the sham group, **p < 0.01. Compared with the model group, #*p* < 0.05, ##*p* < 0.01.

Here, we first analyzed the expression of FABP1 in renal tissues. As shown in Figure 3A–C. Compared with the sham group, the expression of FABP1 in renal tissues of the model group was significantly increased (*p* < 0.01), and the intervention of G-Rg1 decreased the expression of FABP1 in renal tissues of the model (*p* < 0.05). Additionally, Compared with the sham group, the expression of HO-1 was significantly higher in the model group, while the expression of Nrf2 (nucleus) was significantly lower (*p* < 0.01), but G-Rg1 suppressed the expression of HO-1 and promoted the expression of Nrf2 (nucleus) (Figure 3D, *p* < 0.05).

### Effect of G-Rg1 on cell proliferation in hypoxia/reoxygenation-induced cells

**Figure 4.**
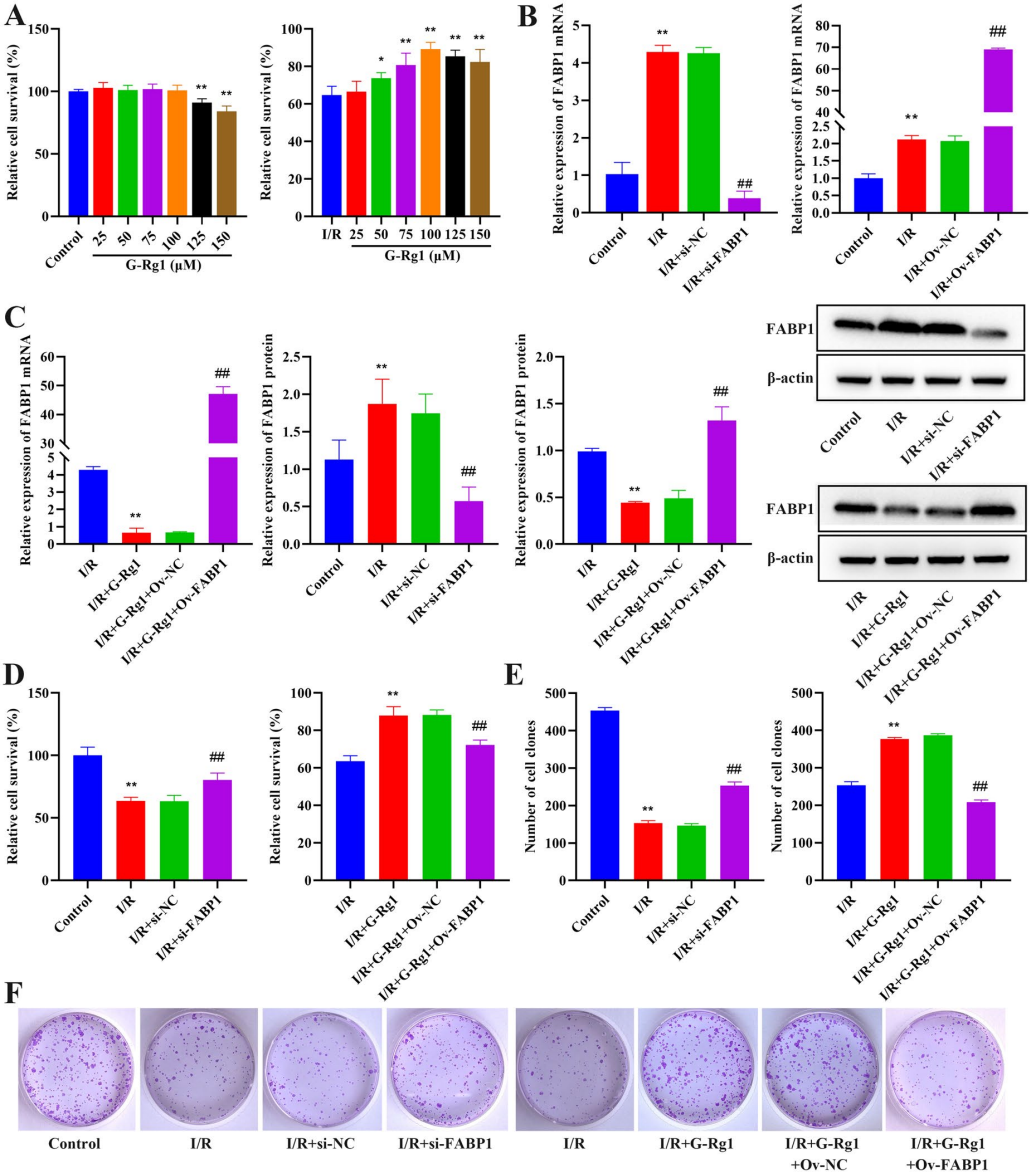
Effect of G-Rg1 on hypoxia/reoxygenation-induced cell proliferation. (A) CCK-8 assay for cell viability. (B) The mRNA expression of FABP1 in cells. (C) The mRNA expression of FABP1 and Western blot results of FABP1 expression in cells, protein band calculated as a ratio relative to β-actin protein levels. (D) CCK-8 assay for cell viability. (E) Cloning assay to detect cell proliferation. (F) Representative images of cloning experiments (crystal violet staining). The data are expressed as the mean ± SD. For the control group, I/R group, I/R + si-NC group, I/R + si-FABP1 group, Compared with the control group, *p < 0.05, **p < 0.01. Compared with the I/R group, ##*p* < 0.01. For the I/R group, I/R + G-Rg1 group, I/R + G-Rg1 + Ov-NC group, I/R + G-Rg1 + si-FABP1 group, Compared with the I/R group, *p < 0.05, **p < 0.01. Compared with the I/R + G-Rg1 group, ##*p* < 0.01.

In this part, we first screened the optimal concentration of G-Rg1, as shown in Figure 4A, when the concentration of G-Rg1 was 100 μM, it was nontoxic to the cells in the control group, which was the maximum nontoxic dose in this experiment (*p* > 0.05). However, the cell survival rate was higher when 100 μM of G-Rg1 treated I/R cells (*p* < 0.01). Therefore, 100 μM of G-Rg1’s was used for subsequent experiments. Following FABP1 silencing, both mRNA and protein expression of FABP1 were significantly reduced. Conversely, FABP1 overexpression resulted in elevated levels of FABP1 (Figure 4B and C, p<0.01). Since the above results showed high expression of FABP1 in model kidney tissues, we analyzed the effect of silencing FABP1 or G-Rg1 + overexpression of FABP1 on cell survival. The results showed that silencing FABP1 increased cell survival (Figure 4D–F, p<0.01), while overexpression of FABP1 inhibited the enhancement of cell survival by G-Rg1 (Figure 4D–F, p<0.01), suggesting that G-Rg1 may improve I/R cell survival by inhibiting FABP1.

### Effect of G-Rg1 on apoptosis, inflammation, and oxidative stress in hypoxia/reoxygenation-induced cells

**Figure 5.**
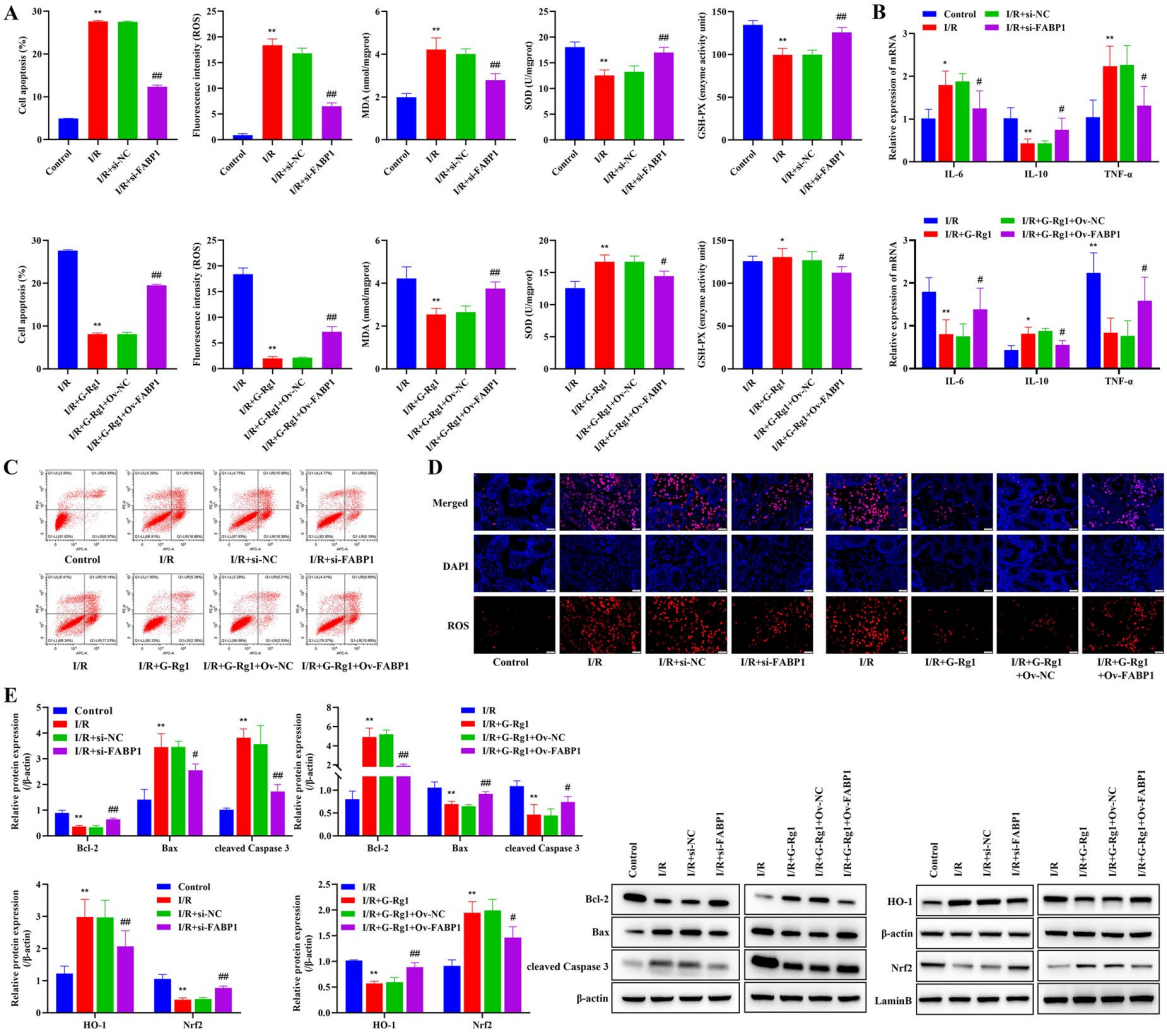
Effect of G-Rg1 on apoptosis, inflammation, and oxidative stress in hypoxia/reoxygenation-induced cells. (A) Apoptosis and levels of ROS, MDA, SOD, and GSH-Px in cells. (B) The mRNA expression of IL-6, IL-10, and TNF-α in cell. (C) Apoptosis flow cytogram. (D) Immunofluorescence staining for ROS in cell (40×, Scale bar, 20 μm). (E) Western blot results of Bcl-2, Bax, cleaved Caspase3, HO-1, and Nrf2 expression in cells, protein band calculated as a ratio relative to β-actin or LaminB protein levels. The data are expressed as the mean ± SD. For the control group, I/R group, I/R + si-NC group, I/R + si-FABP1 group, Compared with the control group, *p < 0.05, **p < 0.01. Compared with the I/R group, ##*p* < 0.01. For the I/R group, I/R + G-Rg1 group, I/R + G-Rg1 + Ov-NC group, I/R + G-Rg1 + si-FABP1 group, Compared with the I/R group, *p < 0.05, **p < 0.01. Compared with the I/R + G-Rg1 group, ##*p* < 0.01.

Also, silencing FABP1 inhibited apoptosis (Figure 5A and C, p<0.01), suppressed the levels of ROS and MDA, increased the activities of SOD and GSH-Px (Figure 5A and D, p<0.01), and attenuated the expression of cytosolic IL-6 and TNF-α (Figure 5B, *p* < 0.05), decreased the expression of Bax, cleaved Caspase 3, and HO-1 in cells (Figure 5E, *p* < 0.05), and promoted cellular IL-10, Bcl-2, and Nrf2 (nucleus) expression (Figure 5B and E, p<0.05). However, overexpression of FABP1 inhibited the inhibitory effect of G-Rg1 on apoptosis, the balance of oxidative stress, and the attenuating effect on the expression of pro-inflammatory cytokines (*p* < 0.05), and G-Rg1 + Ov-FABP1 promoted the expression of HO-1 and suppressed the expression of Nrf2 (nucleus) when compared with the G-Rg1 group (*p* < 0.05), demonstrating that G-Rg1 may inhibit hypoxia/reoxygenation- induced apoptosis, inflammation, and oxidative stress in cells *via* FABP1.

## Discussions

In this study, we found that G-Rg1 reduced the levels of Cr, BUN, NGAL, and KIM-1 in the serum, attenuated renal tissue injury, inhibited pro-inflammatory cytokines, lowered the levels of ROS and MDA, and inhibited the expression of FABP1 and HO-1 in renal I/R mice. In addition, overexpression of FABP1 inhibited the ameliorative effect of G-Rg1 on renal I/R.

Clinically, serum Cr and BUN are the common evaluation indexes of renal function, and most of the Cr values higher than normal values represent renal damage, and Cr can more accurately reflect the damage of renal parenchyma [22]. In the human body, blood BUN is the end product of protein metabolism. Although the measurement of blood BUN level can reflect the glomerular filtration function, BUN will be elevated only when the glomerular filtration function drops to more than half of normal [23]. We evaluated the renal function of each group of mice by detecting the levels of Cr and BUN in serum, and the results showed that the levels of Cr and BUN in the model mice were significantly increased, suggesting that the renal function was significantly impaired after renal I/R. The levels of Cr and BUN were significantly reduced in the mice preconditioned with G-Rg1, which indicated that G-Rg1 had a protective effect against renal I/R injury.

In addition to Cr and BUN, we further demonstrated that G-Rg1 protects renal function after renal I/R injury from the perspective of renal histopathological morphology [24]. Moreover, the KIM-1 protein on the cell membrane surface of the kidney rises significantly when injury occurs, and therefore, KIM-1 is often regarded as a classical marker of renal injury [25]. NGAL is an important marker for early detection of acute kidney injury [26]. To better illustrate the protective effect of G-Rg1 on the kidney, we examined the expression of NGAL and KIM-1 in the kidney, and found that G-Rg1 pretreatment reduced the expression of NGAL and KIM-1 in the kidney tissue. In addition, the process of renal I/R injury leads to the apoptosis of a large number of renal parenchymal cells [27]. In this study, G-Rg1 pretreatment significantly reduced apoptosis in renal tissues. It was demonstrated that G-Rg1 improved renal function after renal I/R injury.

The above experiments have demonstrated that G-Rg1 preconditioning has a favorable therapeutic effect on renal I/R injury, but the specific mechanism is unclear. Oxidative stress represented by ROS generation is a key factor in the occurrence of renal I/R injury [27]. When renal I/R injury occurs, the generated free radicals act on lipids and undergo lipid peroxidation to generate MDA, which produces cytotoxicity [28]. SOD and GSH-PX reflect the ability of the organism’s tissue cells to scavenge oxygen-free radicals [29]. In this study, G-Rg1 pretreatment significantly decreased the levels of MDA and ROS and increased the activities of SOD and GSH-PX in renal I/R-injured mice, demonstrating that G-Rg1 pretreatment inhibits excessive lipid peroxidation. Also, renal I/R injury induces an inflammatory waterfall that exacerbates renal injury, and the increase in pro-inflammatory cytokines IL-6 and TNF-α represents the enhancement of the inflammatory response of the kidney after the kidney’s blood supply is restored [30]. In this experiment, G-Rg1 pretreatment attenuated the expression of IL-6 and TNF-α, suggesting that G-Rg1 inhibits the inflammatory response to renal I/R injury.

Low expression of FABP1 ameliorates oxidative stress in mice with NAFLD [18]. It is noteworthy that previous reports have indicated FABP1 also exerts protective effects against renal oxidative stress [19]. However, this study observed that FABP1 exhibits an adverse role under I/R injury. This discrepancy may stem from the specificity of the injury phase and cellular environment. Nrf2 is an important transcription factor in the body’s resistance to oxidative stress and an important regulator of cellular defense against oxidative stress [31]. HO-1 is an antioxidant enzyme that plays a role in antioxidant, anti-inflammatory, and anti-apoptotic pathways [32]. Studies have reported that the expression of Nrf2 and HO-1 is increased in hypoxia- reoxygenation-injured cells, and nuclear translocation of Nrf2 is inhibited [33]. According to our results, G-Rg1 pretreatment inhibited the expression of FABP1 and HO-1 and promoted the nuclear translocation of Nrf2, suggesting that G-Rg1 could attenuate renal I/R injury by inhibiting FABP1 and increasing the nuclear translocation of Nrf2. Alternatively, renal tubular epithelial cells are the main parenchymal cells of the kidney, which are most prone to ischemic injury compared with other renal units [34], so in this study, renal tubular epithelial cells were used as an *in vitro* research object to create an I/R model. In the *in vitro* experiments, G-Rg1 pretreatment also inhibited the expression of FABP1, reduced the levels of IL-6, TNF-α, ROS, and MDA, and decreased apoptosis. Silencing FABP1 and G-Rg1 pretreatment had similar effects on I/R; however, overexpression of FABP1 inhibited the ameliorative effect of G-Rg1 on I/R.

## Conclusions

In summary, G-Rg1 pretreatment suppressed the levels of Cr and BUN levels, attenuated renal tissue injury, inhibited apoptosis, decreased the levels of pro-inflammatory factors, balanced oxidative stress, inhibited the expression of FABP1 and HO-1, and promoted Nrf2 nuclear translocation in renal I/R injury. In addition, overexpression of FABP1 inhibited the ameliorative effect of G-Rg1 on renal I/R injury, suggesting that G-Rg1 may ameliorate renal I/R injury by inhibiting FABP1. However, there are several limitations to our study. For example, this study did not consider extending the reperfusion endpoint, nor did it observe changes in FABP1 expression at different time points, HO-1 regulation independent of Nrf2. we will carry out further experimental studies to explore the above issues in the future.

## Data Availability

The datasets used and/or analyzed during the current study are available from the corresponding author upon reasonable request.
